# A Cool Draught of Water

**Published:** 1892-11

**Authors:** 


### A COOL DRAUGHT OF WATER.

The Orientals have a simple method of cooling water. They fill a porous earthen jar and by the continual evaporation on the surface the water is soon cooled. Sometimes a heavy cloth is wrapped the jar and kept continually wet. On the hottest day water may be cooled in this way in a few hours' time without the use of ice It is desirable that the jar should stand in a draught to assist evaporation, and that the cloth should be continually wet. The common red clay of which our flower pots are made is formed by South American potters into the most picturesque of water jars, which have a curving handle over the top, and two spouts, a large one by means of which the jar is filled, and a small one by which the water is poured out. These jars are used for cooling water in exactly the same way as the Oriental jars, and this method of obtaining cold water in torrid weather is one of the oldest customs among Orientals, and is found generally among aboriginal people.

Where it is easy to obtain ice, it is not necessary to resort, of course, to any such primitive method as this. A great deal of pretension and folly is shown in the modern ice-water pitcher of plated silver. This elaborate and clumsy contrivance keeps water no longer, and it is doubtful if it keeps it as long, as it may be kept in an ordinary pitcher, providing the latter is covered with a tea-cosey so as to admit no air.

This tea-cosey may be as picturesque and beautiful a piece of embroidery as your needle can make. It may be made from a bit of Oriental needlework, lined with some dainty material which does not readily absorb moisture. All that is necessary is that no air shall penetrate to the pitcher. By means of such contrivance as this a pitcher of ice water may be kept cold all night in a bedroom. As a makeshift, a piece of newspaper, fastened entirely around the pitcher so as to admit no air, will do the same thing, but it is an untidy sort of contrivance and any dainty woman would prefer to substitute a permanent cover, which shall be equally efficacious and not so untidy.

There are many people who do not know how to make a tea-cosey, though it is very simple in contrivance. Cut two semicircles of any material which you may choose for the outside of the cosey. Wad them about twice as thick as a comfortable, but do not pack them. Fasten them together at the curving side by a puff of some material to harmonize with the outside. This makes a hollow cushion which may be slipped over a teapot or a water pitcher, and which will keep the heat or cold within from contact with the outer air.

